# Formulation Design and Cell Cytotoxicity of Curcumin-Loaded Liposomal Solid Gels for Anti-Hepatitis C Virus

**DOI:** 10.1155/2022/3336837

**Published:** 2022-03-07

**Authors:** Helmy Yusuf, Erlyn K. D. D. Novitasari, Ni L. W. Purnami, Adhe W. Mahbub, Retno Sari, Dwi Setyawan

**Affiliations:** Department of Pharmaceutical Sciences, Faculty of Pharmacy, Airlangga University, Jl. Mulyorejo, Surabaya 60115, Indonesia

## Abstract

**Backgrounds:**

Curcumin (CUR) is a low-molecular-weight polyphenolic substance obtained from the tuber part of *Curcuma* species. Anti-inflammatory and anti-hepatitis C virus (HCV) activities have been associated with CUR. However, its poor aqueous solubility and low systemic bioavailability have been the challenges in improving the therapeutic efficacy of curcumin.

**Aim:**

The study aimed to produce CUR-loaded liposomal solid gels as anti-HCV delivery systems. Parameters including the physical characteristics and the cell cytotoxicity properties were evaluated.

**Methods:**

The freeze-drying technique was applied to manufacture the CUR-loaded liposomal solid gels. Scanning electron microscopy (SEM), X-ray diffractometry (XRD), and differential thermal analysis (DTA) were involved to reveal the characteristics of the solid gels. Such characteristics were as follows: the morphology and the microscopic structure of the solid gels, the crystallinity structure of the curcumin, and the thermal properties of the mixtures. Furthermore, their cell cytotoxicity was investigated using a Huh7it cell line.

**Results:**

The SEM images confirmed that curcumin liposomes were intact and trapped in the solid gel matrix. The XRD data showed flat patterns diffractograms of the formulations, confirming the transformation of CUR from crystalline to amorphous form. The DTA thermograms showed a single melting endothermic peak at a higher temperature around 200°C, indicating a single-phase transition of the mixtures. The XRD and DTA data revealed the molecular dispersion of CUR in the developed formulations. The cytotoxicity data provided as cell cytotoxicity 50 (CC_50_) for all formulations were ≥25 mg. These data confirmed that the developed liposomal solid gels were not cytotoxic to Huh7it cell line, indicating that the anti-HCV activity would be through a specific pathway and not by its toxicity.

**Conclusion:**

The CUR-loaded liposomal solid gels exhibited the potential and offered an alternative dosage form to improve the therapeutic efficacy of curcumin as an anti-HCV.

## 1. Introduction

Hepatitis has caused severe liver diseases and affects the worldwide population in which hepatitis C is the most common type responsible for mortality cases [1, 2]. Patients with hepatitis C are given drugs through various routes, including oral and parenteral delivery. Oral is preferred to parenteral for its benefits when it comes to the easy use for patient compliance. However, oral drug delivery systems also face major challenges to address various problems such as poor bioavailability and drug targeting [3]. In most cases, a larger dose is used as compensation to achieve effective plasma drug concentration. However, it tends to increase the incidence of adverse effects relevant to such high doses.

As an alternative, the mucosal routes of administration such as sublingual attract most of the recent interests [4, 5]. The sublingual route offers advantages such as rapid and direct absorption into the systemic circulation following local administration under the tongue and avoiding the first-pass effect; moreover, reduced side effects with the use of lower drug doses and improved patient compliance. These features have made it one of the potential routes of delivery that attract most of the recent studies.

CUR is a natural compound that is poorly water-soluble and poor in permeability, hence categorized as a Biopharmaceutics Classification System (BCS) Class IV substance [6, 7]. CUR exhibits poor gastric absorption and rapid degradation in the gastrointestinal (GI) tract, the factors that made it the major problem for its clinical use as a therapeutic agent [8, 9]. On the other hand, curcumin has been reported as an effective antivirus for hepatitis C (anti-HCV) and used in the treatment of liver diseases [10, 11].

Investigation on the absorption and biodistribution of CUR has been reviewed from several studies. It was reported that CUR formulated in liposomal and nanosuspension forms distributed directly into the liver much higher than other organs such as the spleen and brain [12, 13]. This evidence is promising for the facilitation of the anti-HCV mechanism of CUR through direct interaction with hepatocytes, which then impedes the viral attachment and prevents cell-to-cell transmission [14].

The major challenge in the curcumin development into a dosage form is to overcome the poor bioavailability caused by the lack of water solubility and rapid degradation by acidic hydrolysis. Many studies reported efforts to enhance the stability and absorption of curcumin, including formulation of solid dispersions [15], nanoparticles [16], cyclodextrin complexes [17], micelles [18], and liposomes [19].

Drug-loaded liposomes have been one of the most attractive delivery systems in the formulation development as they are very versatile and offer advantages including high drug encapsulation, minimizing the drug dose and avoiding the side effects, prolonged retention at the target site, protection of drugs from biological environments, and extreme tissue permeability [20, 21]. Liposomes have an amphiphilic structure that self-assembles into a spherical structure in aqueous solutions. Enhanced stability of the drugs is achieved by encapsulation in the nonpolar compartment of the lipid bilayer. Moreover, it also prolongs contact with the mucosal surface due to the adhesive property, which increases drug absorption and bioavailability [22, 23].

Phosphatidylcholine (PC) is one of the widely known liposome-forming phospholipids that have attracted researchers in the last decades [24, 25]. PC is a natural phospholipid that consists of hydrophilic phosphate groups and lipophilic acyl chains. Among many manufacturing techniques, thin-film hydration has been widely used and is the most common to produce drug-loaded liposomes. Thin-film hydration is a simple, continuous technique to produce liposomes from a large variety of phospholipids and is scalable in industrial scale-up for mass production that is prospective for the pharmaceutical industries [26, 27].

Additionally, the use of hydrophilic and mucoadhesive polymers as the entrapping matrix can be useful in retaining the drug-loaded liposomes in the mucosal tissue and facilitating an effective and direct absorption. A polymer that proposes easy wetting as well as bound to mucosal tissue to facilitate the release and absorption of the drug would be advantageous. Hydroxypropyl methylcellulose (HPMC) fits such requirements as a polymer that is hydrophilic and also mucoadhesive [28, 29]. HPMC is soluble in water and at the same time can form a gel with only a small amount of water. It has potencies for the enhanced release and absorption of CUR to the systemic circulation through mucosal tissues.

The presented study aimed to develop liposomal solid gels as an alternative dosage form suitable for sublingual administration of CUR to enhance curcumin's bioavailability. Three different natural phospholipids were used in the CUR-loaded liposomal solid gel formulations. They are soy phosphatidylcholine (SPC), hydrogenated-soy phosphatidylcholine (HSPC), and egg phosphatidylcholine (EPC). Further, the curcumin liposomes were entrapped into the HPMC solid gel and freeze-dried to obtain the dry products. The physical characteristics were studied, including microscopic structures, solid-state characteristics, and thermal properties. Moreover, the cell cytotoxicity using Huh7it cell line was also determined. Huh7it is hepatocyte culture, which was derived from human cells [30]. Hepatocytes are the target of infection by hepatitis C viruses (HCV) in which they are the host cell for the viruses to replicate, thus very relevant to the study.

## 2. Materials and Methods

### 2.1. Materials

CUR, cholesterol (CHOL), and D-*α*-tocopheryl polyethylene glycol 1000 succinate (TPGS), sucrose (SUC), diaminobenzidine (DAB) peroxide staining kit, and dimethyl sulfoxide (DMSO) were purchased from Sigma-Aldrich (Singapore). SPC, EPC, and HSPC were from Lipoid GmbH (Germany). HPMC was obtained from Shin-Etsu (Japan). Chloroform and methanol were of analytical grade and obtained from E Merck (Indonesia). 3-(4,5-Dimethylthiazol-2-yl)-2,5-diphenyltetrazolium bromide (MTT) reagent was from Roche (Germany). Dulbecco's modified Eagle medium (DMEM), high glucose was from GIBCO (United Kingdom). Huh7it cells were kindly provided by the Institute of Tropical Disease, Universitas Airlangga, Surabaya, Indonesia.

### 2.2. Preparation of CUR-Loaded Liposomal Solid Gels

The thin-film hydration technique was selected to produce the liposomes. CUR, phospholipids, CHOL, and TPGS were dissolved in a chloroform/methanol (9 : 1) solvent system to prepare the stock solutions at concentrations of 0.1%w/v, 2%w/v, 0.2%w/v, and 0.05%w/v. Each of these stock solutions was pipetted and mixed up to set a weight ratio of CUR : SPC/HSPC/EPC : CHOL : TPGS (1 : 20 : 2 :  0.5). Afterward, the mixtures were put under vacuum for 2 hours to remove the solvents until a thin-film lipid remained. The lipid film was hydrated for 15 min with 1.0 ml of sucrose solution (10% w/v) pre-heated at 60°C in PBS buffer (pH 7.4) to form the liposomes. The liposomes were settled for 1 hour at room temperature and sonicated for 5 min using a bath sonicator to obtain clearer dispersion. The liposomes were dispersed into 1.0 ml HPMC gel (0.4%w/v) and freeze-dried overnight to obtain solid products.

### 2.3. Scanning Electron Microscopy (SEM)

The morphology of the CUR-loaded liposomal solid gels was examined using SEM. The gel products were gently crushed into small fragments and stocked onto plates of 25 mm in diameter. Plates were assembled to the specimen mounts and further gold-coated by approximately a 5 nm-thick layer. The coated specimens were scanned and image-captured using an electron microscope (TM3000 Tabletop Microscope, Japan).

### 2.4. X-Ray Diffraction Analysis (XRD)

The CUR-loaded liposomal solid gels were evaluated for the crystalline structures of the entrapped CUR using an X-ray diffraction instrument (Philips X'Pert PRO PANalytical, Netherlands). Samples were flatted on the sample holder and scanned with the angle range of 2 theta of 5 to 50° at room temperature. The measurement condition was using a voltage of 40 kV and 40 mA.

### 2.5. Differential Thermal Analysis (DTA)

The thermal properties of the CUR-loaded liposomal solid gels were determined using a DTA instrument (Mettler Toledo FP 85, Switzerland). A small amount of sample was loaded into an aluminum crucible pan and crimped. Scanning was performed using a heating rate of 10°C/min from 30°C to 300°C. The samples were analyzed for the phase transition temperature, indicated by the detected endothermic or exothermic peaks. The experiments were performed in triplicate.

### 2.6. Cell Cytotoxicity

The MTT (Roche, Germany) assay was employed in the cell cytotoxicity study by determining the viability of Huh7it cell line after treated with the formulations [30, 31]. Huh7it cells were incubated for 48 hours with a dilution series of CUR-loaded liposomal solid gels or medium as a control in 96-well plates. After the incubation, MTT reagent was added at a volume of 10 *μ*l to each well. Cells were incubated for another 4 hours at 37°C. The assay quantified the amount of formazan as a result of converted MTT by mitochondrial dehydrogenases. The quantity of the formed formazan correlates with the number of living cells, which then reflects the viability of the cells. The reaction was stopped by removing the supernatant and replaced by the addition of 100 *μ*L DMSO. The absorbance of formazan in each well was measured at 550 nm using a microplate reader (EL808 Microplate Reader, BioTek Instruments, USA). Percent cell viability was calculated for each dilution of the gels-containing curcumin liposomes as compared to the control. Measurements were made in triplicate.

## 3. Results and Discussion

### 3.1. Morphology of CUR-Loaded Liposomal Solid Gels

**Figure 1:**
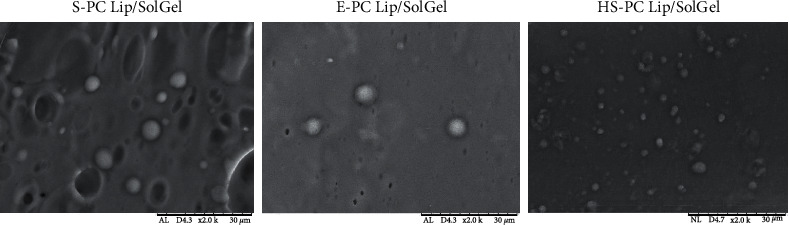
Morphology of the CUR-loaded liposomal solid gels obtained by SEM at a magnification level of 2.000x. S-PC Lip/SolGel, soy-phosphatidylcholine liposomal solid gel; E-PC Lip/SolGel, egg-phosphatidylcholine liposomal solid gel; HS-PC Lip/SolGel, hydrogenated-soy-phosphatidylcholine liposomal solid gel.

The SEM studies were applied to disclose the morphology and intactness of the liposomes entrapped inside the solid gel matrix. A single SEM magnification was used at the level of 2.000x to observe the thorough morphology of the developed liposomal solid gels and to further inspect the intactness of the entrapped liposomes. The whole morphology of the CUR-loaded liposomal solid gels is illustrated in Figure 1. The solid gels appear subtle, hollow, and slightly porous with the spherical liposomes distributed around. Nevertheless, some regions show a coarser microstructure where the liposomes were scattered in the matrix. For instance, the morphology of S-PC Lip/SolGel showed a relatively more porous solid gel matrix than those of E-PC Lip/SolGel and HS-PC Lip/SolGel. With the same manufacturing technique, the liposomes prepared using the SPC, EPC, and HSPC showed similar morphology that was spherical and homogeneously distributed in the porous solid gel matrix. Such porous structure might be initiated by the entrapped ice crystals that sublimated during the freeze-drying process and resulted in a hollow structure [32, 33]. The entrapped liposomes were a few micrometers in size, which indicates that they aggregated during the incorporation into the solid gel matrix. Yet, the liposomes were intact and well preserved in the matrix. Moreover, the curcumin crystals were not observed in the SEM images for they might be molecularly distributed within the liposomal membrane.

### 3.2. Crystallinity

**Figure 2:**
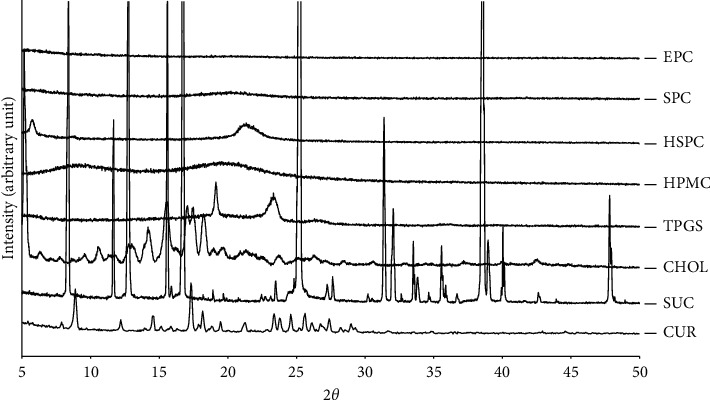
XRD diffractograms of all single components used in the developed CUR-loaded liposomal solid gels. Samples were scanned within a range of reflecting angle 2*θ* of 5 to 50° at room temperature. CUR, curcumin; SUC, sucrose; CHOL, cholesterol; TPGS, D-*α*-tocopheryl polyethylene glycol 1000 succinate; HPMC, hydroxypropyl methylcellulose; HSPC, hydrogenated-soy phosphatidylcholine; SPC, soy phosphatidylcholine; EPC, egg phosphatidylcholine.

The degree of crystallinity of the entrapped CUR and other excipients used in the developed liposomal solid gel formulations was studied using X-ray diffraction analysis. The integrated intensity of sample reflection is measured within a range of reflecting angle 2*θ*. XRD patterns of all materials alone used in the formulation are shown in Figure 2. The XRD pattern of CUR alone reveals intense peaks within the range of measured 2*θ* angles, which confirm its crystalline nature [34]. Other components including SUC, CHOL, and TPGS also displayed similar patterns where their intensive peaks indicated the crystalline form. In contrast, the three phospholipids, i.e., SPC, EPC, and HPMC, showed flat patterns, indicating their noncrystalline nature [35, 36], whilst HSPC showed few peaks, indicating its liquid crystalline nature powder [37]. This liquid crystal nature is suitable for such bioavailability enhancement strategy of poorly permeable drugs [38].

**Figure 3:**
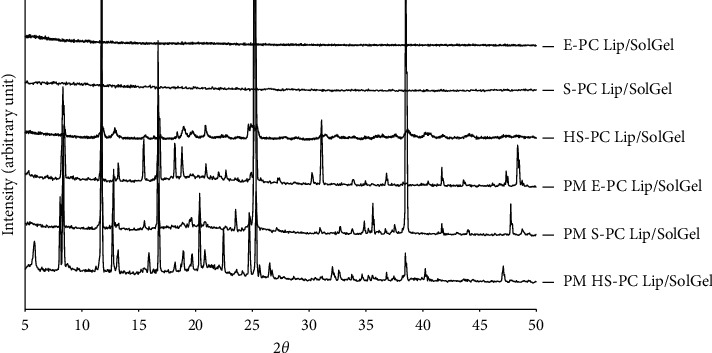
XRD diffractograms of CUR-loaded liposomal solid gel formulations. Samples were scanned within a range of reflecting angle 2*θ* of 5 to 50° at room temperature. E-PC Lip/SolGel, egg-phosphatidylcholine liposomal solid gel; S-PC Lip/SolGel, soy-phosphatidylcholine liposomal solid gel; HS-PC Lip/SolGel, hydrogenated-soy-phosphatidylcholine liposomal solid gel; PM, the physical mixture of each formulation.

Conversely, the diffractogram also revealed the disappearance and diffusion of those utmost peaks of the three CUR-loaded liposomal solid gels: S-PC Lip/SolGel, E-PC Lip/SolGel, and HS-PC Lip/SolGel (Figure 3). SPC and EPC showed similar character as liposomal constituents, where they share a similar capability in encapsulating CUR inside their lipid bilayer. Only HS-PC Lip/SolGel showed a slightly different pattern, where small peaks were still detected, indicating a more ordered mixture. However, those peaks were significantly reduced as compared to the corresponded physical mixture, implying a significant decrease of CUR crystallinity. These results implied that CUR was molecularly dispersed in the liposomal solid gels. Amorphization of the CUR provides several advantages compared to its crystalline counterpart, comprising improved solubility and enhanced drug dissolution rate. The diffractograms of PM S-PC Lip/SolGel, PM E-PC Lip/SolGel, and PM HS-PC Lip/SolGel disclosed that the intense peaks were retained, indicating the still existence of CUR in crystalline form (Figure 3).

### 3.3. Thermal Properties

**Figure 4:**
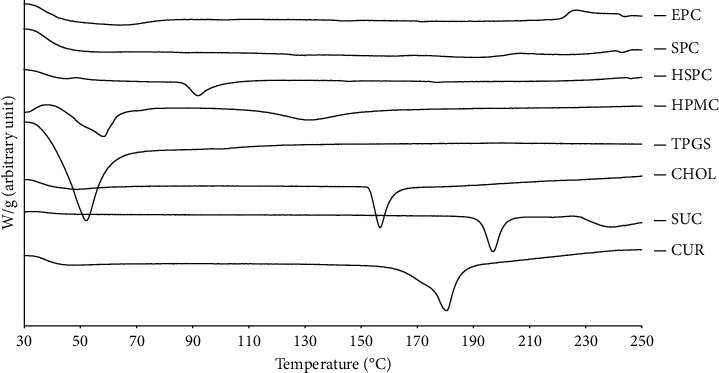
DTA thermograms of all single components used in the developed CUR-loaded liposomal solid gels. Samples were scanned within a range of 30 to 250°C, at heating rate of 10°C/min. CUR, curcumin; SUC, sucrose; CHOL, cholesterol; TPGS, D-*α*-tocopheryl polyethylene glycol 1000 succinate; HPMC, hydroxypropyl methylcellulose; HSPC, hydrogenated-soy phosphatidylcholine; SPC, soy-phosphatidylcholine; EPC, egg-phosphatidylcholine.

DTA was used to characterize the thermal properties in terms of their phase behavior and the miscibility of the obtained liposomal solid gels. DTA is selected to reveal the degree of crystallinity that corresponded with phase transitions of the compounds as a function of temperature. The analysis was carried out to all single components as well as their mixture in the developed solid gel systems. CUR exhibited a sharp endothermic peak at 174°C, similar to TPGS, CHOL, and SUC that showed similar sharp endothermic peaks at 40°C, 150°C, and 195°C, respectively (Figure 4).

All the sharp peaks were conforming to their melting point indicating their crystalline nature. The DTA thermogram of HPMC showed broader endothermic peaks at 52°C and 125°C, which is indicative of a less ordered structure of the polymer solid state. SPC and EPC exhibited wide endothermic peaks that were hard to define [36]. It is speculated that their phase transition is too broad that the applied heating rate (10°C/min) was not sufficient to record the event. It is suggested that a slower heating rate can be applied to obtain reliable phase transition from this type of phospholipids [39]. Nevertheless, these DTA thermograms of the single component supported the results of XRD studies.

**Figure 5:**
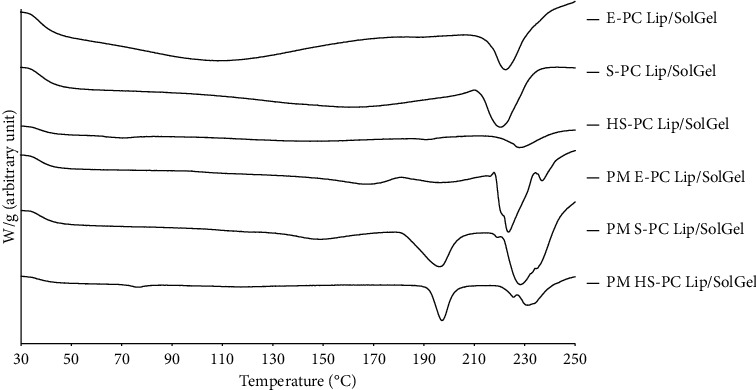
DTA thermograms of CUR-loaded liposomal solid gel formulations. Samples were scanned within a range of 30 to 250°C, at a heating rate of 10°C/min. E-PC Lip/SolGel, egg-phosphatidylcholine liposomal solid gel; S-PC Lip/SolGel, soy-phosphatidylcholine liposomal solid gel; HS-PC Lip/SolGel, hydrogenated-soy-phosphatidylcholine liposomal solid gel; PM, the physical mixture of each formulation.

CUR-loaded liposomal solid gels showed a single melting endothermic peak at a higher temperature of 210°C for S-PC Lip/SolGel and 215°C for E-PC Lip/SolGel, while two peaks were observed at 191°C and 225°C for HS-PC Lip/SolGel (Figure 5). It is self-suggestive that the existence of liposomal solid gels was in a more ordered form with behavior like crystalline form. The SPC, EPC, and HSPC liposomal constituents were not different in their thermal properties. None of the thermograms of these three formulations indicated a single endothermic peak of curcumin, suggesting that it was not phase-separated in the mixtures. Another peaks associated with the other ingredients were also absent, indicating ample amorphization when formulated as solid gels. These DTA results were consistent with the XRD data.

The thermogram of PM S-PC Lip/SolGel showed three peaks at 150°C, 190°C, and 220°C. These were similar to the PM E-PC Lip/SolGel that showed three peaks at 170°C, 190°C, and 215°C. These three peaks are associated with cholesterol, curcumin, and sucrose, respectively. The PM HS-PC Lip/SolGel also revealed two peaks at 190°C and 225°C that corresponded to the phase-separated curcumin and sucrose in the mixtures. All PM thermograms exhibited peaks of almost every single component, suggesting that a simple mixing is not sufficient to obtain homogenous distribution of the liposomes and molecular dispersion of the CUR in the solid gels.

### 3.4. Cell Cytotoxicity

Cell cytotoxicity was determined by the MTT assay to investigate whether the anti-HCV mechanism is initiated by the toxicity or other pathways. The determination was based on the concentration of the samples at which cell viability is reduced by 50% compared to the control cell group. The data are provided as cell cytotoxicity 50 (CC_50_) of the CUR-loaded liposomal solid gels, which are summarized in Table 1. There may be no cellular toxicity for cells treated with CUR-loaded liposomal solid gels at the applied concentration range as revealed by the MTT assay. S-PC Lip/SolGel and E-PC Lip/SolGel were found to be less cytotoxic (>25 × 10^3^ *μ*g/mL) than HS-PC Lip/SolGel (25 × 10^3^ *μ*g/mL). The SPC, EPC, and HSPC together with the other excipients were proven not toxic to cells. Similar studies reported that the CC_50_ of tested CUR materials using the Huh7it cell lines were >85 *μ*g/mL [30] and >100 *μ*g/mL [11]. Moreover, the CC_50_ of ribavirin, an antiviral drug, used as the positive control in the toxicity study has been reported at >50 *μ*g/mL using the same Huh7it cell line [11]. Based on these cytotoxicity data, the developed liposomal solid gel delivery systems showed less toxicity than the ribavirin. These results indicated that all materials included in the formulations were not toxic to the normal cells. Hence, it is predicted that the anti-HCV effects of CUR-loaded liposomal solid gels may not be developed through its cellular toxicity; rather it may utilize a specific pathway. Further investigation on this particular topic is on the future direction of the study.

The present study revealed that incorporating CUR in liposomal solid gels is advantageous. Encapsulation into liposomal phospholipids and entrapment into a polymeric matrix have transformed the CUR from crystalline to become amorphous, which is significant for solubilization and absorption enhancement into the systemic circulation. Furthermore, the liposomal solid gels, developed for mucosal route administrations such as sublingual, may also be an excellent strategy to overcome the bioavailability-related problems of CUR. Such route allows direct absorption to the systemic circulation and further delivers CUR to the liver at a higher concentration, as reported in many studies [12]. It is a promising anti-HCV therapy concerning the target cells are hepatocytes as the majority of the parenchymal cells found in the liver.

Further investigations are directed to study the performance of the developed delivery systems in another aspect such as the therapeutic efficacy as anti-HCV and emphasis on the specific mechanism of the anti-HCV activity. Several characterizations including morphology image of the cells after the treatment and quantitative analysis using flow cytometry are essential. Investigation of biodistribution of the developed delivery systems is also worth investigating. Moreover, further development into a sublingual tablet formulation is another interesting aspect. Selection of the manufacturing and other drying techniques is interesting to provide insights for the optimum manufacturing procedures.

## 4. Conclusions

In the present study, CUR-loaded liposomal solid gels were successfully prepared using the freeze-drying technique. The improvement in the physical characteristics of CUR-loaded liposomal solid gels could be attributed to amorphization and dispersion at a molecular level of CUR in the liposomal solid gels as disclosed by SEM, XRD, and DTA data. Furthermore, the prepared liposomal solid gels of CUR are not cytotoxic, which is potential for an anti-HCV delivery system. Therefore, the CUR-loaded liposomal solid gels offer benefits to improve biopharmaceutical characteristics of CUR that potentiate its therapeutic efficacy in HCV infection.

## Figures and Tables

**Table 1:** Cell cytotoxicity (CC_50_) of CUR-loaded liposomal solid gels, determined by the incubation of a series of sample concentrations to reach 50% of cells that remain viable.

Concentration (*μ*g/mL)	Viability (%)		
	S-PC Lip/SolGel^a^	E-PC Lip/SolGel^b^	HS-PC Lip/SolGel^c^
0.7 × 10^3^	96.1 ± 0.04	95.7 ± 0.01	97.7 ± 0.01
1.5 × 10^3^	95.6 ± 0.01	95.0 ± 0.01	97.3 ± 0.02
3.0 × 10^3^	92.3 ± 0.01	92.3 ± 0.02	96.8 ± 0.002
6.0 × 10^3^	90.5 ± 0.01	91.7 ± 0.03	93.5 ± 0.01
12.0 × 10^3^	88.8 ± 0.01	85.9 ± 0.02	85.9 ± 0.01
25.0 × 10^3^	81.3 ± 0.05	73.9 ± 0.02	46.3 ± 0.01

Notes: ^a^CC_50_ = 79 × 10^3^ *μ*g/ml; ^b^CC_50_ = 52 × 10^3^ *μ*g/ml; ^c^CC_50_ = 25 × 10^3^ *μ*g/ml. CC_50_ was calculated by extrapolating the percent viability data at 50% to the corresponding concentration. Abbreviations: S-PC Lip/SolGel. soy phosphatidylcholine liposomal solid gel; E-PC Lip/SolGel. egg phosphatidylcholine liposomal solid gel; HS-PC Lip/SolGel. hydrogenated-soy phosphatidylcholine liposomal solid gel.

## Data Availability

Data used in this study are available from the corresponding author on request.
